# Guselkumab Safety in Patients With Latent Tuberculosis: Analysis of 11 Studies in Psoriatic Disease

**DOI:** 10.1111/1346-8138.70314

**Published:** 2026-06-01

**Authors:** Luis Puig, Tsen‐Fang Tsai, Enrique R. Soriano, Tina Bhutani, Megan Miller, Alexa P. Kollmeier, Shu Li, Hewei Li, Sarah Ofori, Jeffrey Pawlikowski, Elizabeth Adamson, Melinda Gooderham, Angela Londoño Garcia, Mark Lebwohl, Peter Nash

**Affiliations:** ^1^ Hospital de la Santa Creu i Santa Pau, Universitat Autònoma de Barcelona Barcelona Spain; ^2^ National Taiwan University Hospital Taipei Taiwan; ^3^ Rheumatology Section, Internal Medicine Service Hospital Italiano de Buenos Aires, and University Hospital Italiano de Buenos Aires Buenos Aires Argentina; ^4^ Synergy Dermatology San Francisco California USA; ^5^ Johnson & Johnson Spring House Pennsylvania USA; ^6^ Johnson & Johnson San Diego California USA; ^7^ Johnson & Johnson Titusville New Jersey USA; ^8^ Johnson & Johnson Horsham Pennsylvania USA; ^9^ SKiN Centre for Dermatology, Department of Medicine at Queen's University, and Probity Medical Research Peterborough Ontario Canada; ^10^ CES University Medellín Colombia; ^11^ Icahn School of Medicine at Mount Sinai New York New York USA; ^12^ Griffith University School of Medicine, Brisbane, and Queensland Health, Sunshine Coast University Hospital Brisbane Queensland Australia

**Keywords:** guselkumab, interleukin‐23 inhibitors, psoriasis, psoriatic arthritis, tuberculosis

## Abstract

Certain psoriatic disease treatments, including tumor necrosis factor‐α inhibitors (TNFi), increase tuberculosis (TB) risk. Testing for latent TB infection (LTBI) before initiating systemic therapy has been standard practice for psoriasis (PsO) patients; however, updated consensus states routine testing is not required before initiating interleukin (IL)‐17 or IL‐23 inhibitors. This analysis reports safety outcomes in LTBI+ patients with moderate‐to‐severe PsO or active psoriatic arthritis (PsA) who received guselkumab for up to 5 years. Data were pooled from 11 phase 2/3 studies (7 PsO, 4 PsA). Guselkumab 100 mg was generally administered at Week 0, Week 4, then every 8 weeks (PsO studies) or every 4 or 8 weeks (PsA studies). Placebo‐randomized patients crossed over to guselkumab at Week 16 and Week 24 in PsO and PsA studies, respectively. Patients with active TB or a history of active TB were excluded. LTBI+ patients could participate if LTBI treatment was initiated before/with the first dose of study drug or if LTBI treatment was completed within 5 years before the first dose of study drug. Among 5 255 randomized patients, 374 (7.1%) had LTBI and received preventive treatment, most commonly isoniazid (82.1%) and rifampicin (11.8%). No new‐onset TB or LTBI reactivation occurred in any guselkumab‐treated patient. Through Year 1, liver transaminase elevations were more common in LTBI+ vs. LTBI− patients; ≤ 2% of LTBI+ patients had CTCAE Grade 3 elevations vs. 0.5% of LTBI− patients (no LTBI+ patients had Grade 4 elevations). From Year 1–5 (after ~98% of LTBI+ patients completed preventive treatment), transaminase elevations were generally similar among LTBI+ and LTBI− patients. The absence of observed TB risk in guselkumab‐treated patients suggests IL‐23 inhibitors may be better treatment options than TNFi in high‐risk patients, including those in TB‐endemic regions.

## 1 Introduction

Worldwide, tuberculosis (TB) is the leading cause of death from a single infectious agent, causing > 1.2 million deaths/year. Despite efforts to increase access to testing and treatments, ~25% of the global population is infected with TB, and 5%–10% of those infected will develop active disease during their lifetime [1, 2, 3, 4]. Certain psoriatic disease treatments, including tumor necrosis factor‐α (TNF) and Janus kinase (JAK) inhibitors, increase TB risk, as TNF and JAKs play integral roles in formation, function, and maintenance of TB granulomas (the body's main defense against *Mycobacterium tuberculosis* ). Therefore, guidelines have recommended screening patients for latent TB infection (LTBI) before starting any biologic treatment for psoriatic disease and prescribing preventive treatment for LTBI+ individuals [2, 3, 4]. However, several analyses have reported results suggesting that treatments that inhibit interleukin (IL)‐17 or IL‐23 do not increase LTBI reactivation risk [2, 3, 4, 5, 6, 7, 8]. Based on a comprehensive review of available evidence, the National Psoriasis Foundation (NPF) and International Psoriasis Council (IPC) have recently issued a position statement that routine LTBI screening is not required prior to or during treatment of psoriasis (PsO) with IL‐17 or IL‐23 inhibitors [9].

Here, we report safety outcomes in LTBI+ patients who received guselkumab for moderate‐to‐severe PsO or active psoriatic arthritis (PsA) for up to 5 years.

## 2 Methods

Safety data were pooled from 11 phase 2 and 3 studies of guselkumab in psoriatic disease (7 PsO and 4 PsA studies) [10]. Nine studies were placebo‐controlled, and five included active comparisons to adalimumab, secukinumab, or ustekinumab [10, 11, 12, 13, 14]. Guselkumab was generally administered as 100‐mg subcutaneous injections at Week 0, Week 4, then every 8 weeks in PsO studies and every 4 or 8 weeks in PsA studies. Patients randomized to placebo crossed over to guselkumab at Week 16 and 24 in the PsO and PsA studies, respectively.

All patients were screened for TB at baseline. Patients with active TB or a history of active TB (regardless of previous treatment) were excluded. LTBI+ patients could participate if appropriate LTBI treatment was initiated prior to or with the first dose of study drug or if appropriate treatment was completed within 5 years before the first dose of study drug. As some TB treatments increase risk of hepatotoxicity [2, 3, 4], liver transaminases (alanine aminotransferase [ALT] and aspartate aminotransferase [AST]) were measured at prespecified timepoints in all 11 studies, generally every 4–8 weeks through Week 24, every 4–12 weeks through 1 year, and every 16–24 weeks for up to 5 years.

## 3 Results

Among 5 255 randomized patients, 374 (7.1%) had LTBI and received preventive treatment. LTBI prevalence was highest among patients in the Asia‐Pacific region (10.0%), followed by Eastern Europe (7.3%), Western Europe (7.3%), and North America (5.3%). The mean (standard deviation) age of LTBI+ patients was 49.7 (12.0) years, and most patients were male (67.1%) and White (76.7%); these characteristics were generally similar to those for the full pooled patient population across all 11 studies [10].

LTBI treatment was initiated before (88.2%), with (6.1%), or after (5.6%) the first dose of study drug (median, −8.0 days; interquartile range [IQR], −20.0 to −2.0). Median (IQR) LTBI treatment duration was 185 (124–274) days (Table 1).

**TABLE 1:** Timing of initiation and duration of LTBI treatment by treatment group at randomization.

TB treatment characteristics	Placebo	Guselkumab	Adalimumab	Ustekinumab	Secukinumab	Total
Patients treated with TB medication, *n*	71	213	43	14	33	374
Median (IQR) time of initiation from first dose, days	−13.0 (−25; −5)	−9.0 (−21; −3)^a^	−3.5 (−9; 0)^a^	−1.5 (−8; 0)	−6.0 (−19; −2)	−8.0 (−20; −2)^a^
Prior to the first dose of the study drug, *n* (%)						
1 week prior to first dose	21 (29.6%)	79 (37.1%)	18 (41.9%)	5 (35.7%)	14 (42.4%)	137 (36.6%)
2 weeks prior to first dose	12 (16.9%)	41 (19.2%)	8 (18.6%)	3 (21.4%)	3 (9.1%)	67 (17.9%)
4 weeks prior to first dose	23 (32.4%)	33 (15.5%)	2 (4.7%)	1 (7.1%)	4 (12.1%)	63 (16.8%)
8 weeks prior to first dose	4 (5.6%)	18 (8.5%)	2 (4.7%)	0	3 (9.1%)	27 (7.2%)
> 8 weeks prior to first dose	7 (9.9%)	23 (10.8%)	2 (4.7%)	0	4 (12.1%)	36 (9.6%)
On first dose day, *n* (%)	1 (1.4%)	10 (4.7%)	5 (11.6%)	5 (35.7%)	2 (6.1%)	23 (6.1%)
After first dose of study drug, *n* (%)	3 (4.2%)	9 (4.2%)	6 (14.0%)	0	3 (9.1%)	21 (5.6%)^b^
Patients with duration of LTBI treatment data available, *n*	62	191	34	11	24	322
Median duration (IQR), days	185.5 (154; 265)	185 (142; 275)	215 (174; 262)	269 (102; 274)	122 (95.5; 226)	185 (124, 274)

Abbreviations: IQR, interquartile range; LTBI, latent tuberculosis infection; TB, tuberculosis.

^a^ Guselkumab, *n* = 209; adalimumab, *n* = 42; total, *n* = 369.

^b^ Of the 21 patients who started LTBI treatment after initiating study drug, 13 were QuantiFERON Gold positive (QFT+) at baseline, and 1 had a pulmonologist evaluation that could not rule out LTBI at baseline; these patients did not start LTBI treatment as required and were therefore classified as major protocol deviations. Six patients were QFT− at baseline but received LTBI treatment during the study (1 guselkumab‐treated patient received Rifabutin for *Heliobacter pylori*; 1 guselkumab‐treated patient received rifampicin for an infection following total knee replacement; 1 guselkumab‐treated patient who worked in a hospital developed a QFT+ test during the study, active TB was ruled out, and LTBI treatment was initiated; 1 adalimumab‐treated patient initiated LTBI treatment during the trial; 1 adalimumab‐treated patient developed new‐onset active TB and was treated accordingly; and 1 secukinumab‐treated patient came in contact with a person with active TB during the study, tested QFT+, and started LTBI treatment). One additional placebo‐randomized patient with missing QFT results at baseline received LTBI treatment prior to discontinuing the study after Week 3.

The most commonly prescribed LTBI treatments were isoniazid (82.1%) and rifampicin (11.8%). Most patients received one TB medication (89.8%); 9.4% received two, 0.5% received three, and 0.3% received four medications.

No cases of new‐onset active TB or LTBI reactivation were observed among guselkumab‐treated patients (*N* = 4 339; 10 787 patient‐years). During the placebo‐controlled period, similar proportions of patients in the guselkumab and placebo groups, respectively, experienced adverse events (AEs) in the LTBI+ (47.9% vs. 46.5%) and LTBI– (48.3% vs. 48.0%) cohorts; serious AE rates were also similar (LTBI+: 2.1% vs. 2.8%; LTBI–: 2.0% vs. 2.3%). Through the end of follow‐up, guselkumab‐treated LTBI+ and LTBI– patients had similar cumulative rates of AEs (72.5% vs. 74.6%) and serious AEs (12.1% vs. 10.1%), respectively (Table 2). Across all 11 studies, two serious cases of new‐onset active TB were reported in LTBI− patients who received adalimumab [7].

**TABLE 2:** Safety in guselkumab‐treated LTBI+ and LTBI− patients.

	Placebo‐controlled period				Through the end of follow‐up	
	Placebo^a^		Guselkumab		All guselkumab^b^	
	LTBI+	LTBI−	LTBI+	LTBI−	LTBI+	LTBI−
Treated patients, *n*	71	990	188	2 069	313	4 086
Mean duration of follow‐up, weeks	19.6	19.4	20.2	19.8	129.0	127.9
Mean number of treatment administrations	6.8	7.4	7.0	7.2	24.9^c^	24.2^c^
Total patient‐years of follow‐up	26.7	368.0	72.7	783.4	773.6	10 013.9
Median PY of follow‐up	0.3	0.3	0.4	0.3	2.1	1.7
Patients with active TB, *n*	0	0	0	0	0	0
Patients with ≥ 1 AE, *n* (%)	33 (46.5%)	475 (48.0%)	90 (47.9%)	999 (48.3%)	227 (72.5%)	3048 (74.6%)
Patients with ≥ 1 serious AE, *n* (%)	2 (2.8%)	23 (2.3%)	4 (2.1%)	42 (2.0%)	38 (12.1%)	414 (10.1%)
Number of AEs per 100 PY of follow‐up	123.5	129.1	123.8	127.5	29.3	30.4
Number of serious AEs per 100 PY of follow‐up	7.5	6.2	5.5	5.4	4.9	4.1

Abbreviations: AE, adverse event; LTBI, latent tuberculosis infection; PY, patient‐years; TB, tuberculosis.

^a^ Includes data prior to guselkumab exposure in placebo‐treated patients who later crossed over to guselkumab.

^b^ Includes patients originally randomized to placebo or adalimumab at baseline who crossed over and were treated with guselkumab.

^c^ LTBI+, *n* = 310; LTBI−, *n* = 3 909.

Through Year 1, liver transaminase elevations were more common among LTBI+ vs. LTBI– patients (Table 3). From Year 1 to Year 5 (after ~98% of LTBI+ patients completed preventive treatment), liver transaminases were generally similar in the LTBI+ and LTBI– groups.

**TABLE 3:** Guselkumab‐treated patients with elevated ALT and AST by year.

	Through year 1		Year 1 through year 2		Year 2 through year 3		Year 3 through year 4		Year 4 through year 5	
Patients with increased ALT, *n* (%)^a^										
	LTBI+	LTBI—	LTBI+	LTBI—	LTBI+	LTBI—	LTBI+	LTBI—	LTBI+	LTBI—
*N*	296	3 846	238	3 128	155	1 798	93	1 096	83	1 048
CTCAE Grade 1	114 (38.5%)	1232 (32.0%)	53 (22.3%)	702 (22.4%)	29 (18.7%)	330 (18.4%)	18 (19.4%)	234 (21.4%)	20 (24.1%)	194 (18.5%)
CTCAE Grade 2	10 (3.4%)	74 (1.9%)	1 (0.4%)	31 (1.0%)	1 (0.6%)	16 (0.9%)	0	9 (0.8%)	2 (2.4%)	3 (0.3%)
CTCAE Grade 3	6 (2.0%)	20 (0.5%)	1 (0.4%)	8 (0.3%)	0	1 (0.1%)	1 (1.1%)	1 (0.1%)	0	3 (0.3%)
CTCAE Grade 4	0	0	0	0	0	0	0	0	0	0
Patients with increased AST, *n* (%)^a^										
*N*	296	3 846	237	3 117	155	1 792	93	1 093	82	1 046
CTCAE Grade 1	93 (31.4%)	849 (22.1%)	38 (16.0%)	412 (13.2%)	17 (11.0%)	189 (10.5%)	15 (16.1%)	120 (11.0%)	12 (14.6%)	95 (9.1%)
CTCAE Grade 2	9 (3.0%)	69 (1.8%)	0	24 (0.8%)	1 (0.6%)	7 (0.4%)	1 (1.1%)	3 (0.3%)	1 (1.2%)	3 (0.3%)
CTCAE Grade 3	5 (1.7%)	20 (0.5%)	1 (0.4%)	11 (0.4%)	2 (1.3%)	4 (0.2%)	0	3 (0.3%)	0	3 (0.3%)
CTCAE Grade 4	0	1 (< 0.1%)	0	0	0	0	0	0	0	0

Abbreviations: ALT, alanine aminotransferase; AST, aspartate aminotransferase; CTCAE, United States National Cancer Institute Common Terminology Criteria for Adverse Events; LTBI, latent tuberculosis infection.

^a^ Results do not include data from the psoriasis Phase 2 X‐PLORE study, which did not report CTCAE toxicity grading.

## 4 Discussion

Results of this analysis add to the growing body of evidence supporting the absence of a TB safety signal among patients treated with guselkumab for psoriatic disease [2, 5, 6, 7, 8, 9]. Most recently, the NPF‐IPC position statement provided a comprehensive evaluation of data from clinical trials and real‐world experience with these drugs, including an analysis of > 96 000 reports to the FDA Adverse Event Reporting System (FAERS), which found zero cases of extra‐pulmonary internal organ TB and zero cases of disseminated TB in patients exposed to IL‐17 or IL‐23 inhibitors [9]. This review resulted in a new recommendation that LTBI testing is not required for patients with psoriasis who are initiating treatment with an IL‐17 or IL‐23 inhibitor, except in certain circumstances (e.g., in patients receiving concomitant immunosuppressants that increase TB risk and in geographic areas where TB is endemic) [9].

The NPF‐IPC FAERS database analysis confirmed a signal for increased TB risk with TNF inhibitors, [9] and de novo TB is known to occur in patients receiving these therapies [15]. These findings are consistent with the current analysis of guselkumab trials, which identified 2 cases of new‐onset active TB in adalimumab‐treated patients, compared with zero new‐onset active TB or LTBI reactivation in patients treated with guselkumab or active comparators that inhibit IL‐17 or IL‐12/23.

This is the largest and longest study to date of TB risk in guselkumab‐treated patients, including > 300 patients with LTBI at baseline and > 4 000 patients exposed to guselkumab for up to 5 years (~11 000 patient‐years). The long duration of follow‐up is a strength of this analysis, since LTBI reactivation is relatively uncommon [2]. Consistent with findings from this analysis, the recently completed GUIDE, VISIBLE, and SPECTREM phase 3b PsO studies of guselkumab also reported no cases of new‐onset TB or LTBI reactivation (data on file).

In guselkumab trials, it is possible that TB treatment prevented LTBI reactivation in some patients. However, real‐world psoriasis studies of IL‐23 inhibitors have shown no increased risk of LTBI reactivation in patients who did not receive preventive treatment, often due to safety concerns, including hepatotoxicity and drug–drug interactions [2, 5]. These concerns are heightened when isoniazid is prescribed concomitantly with methotrexate or rifampicin (hepatotoxicity) or when rifampicin is combined with corticosteroids (other drug–drug interactions) [4]. Consistent with these concerns, elevated transaminases were more common in LTBI+ patients than in LTBI– patients through Year 1, when LTBI+ patients were receiving preventive treatment, predominantly with isoniazid (82%) or rifampicin (12%). Rates of transaminase elevations in LTBI+ patients decreased after Year 1, when preventive treatment regimens were complete, suggesting preventive treatment as the most likely cause of transaminase elevations.

Another concern when considering preventive treatment is that improper use can increase the risk of multidrug‐resistant TB. Furthermore, TB screening can delay initiation of treatment for psoriatic disease. For those who test positive for LTBI, guidelines recommend waiting 1 month after starting preventive treatment before initiating biologics [3, 4, 6].

## 5 Conclusions

Results across the guselkumab psoriatic disease clinical development program have identified no cases of new‐onset active TB or LTBI reactivation in up to 5 years of treatment. The absence of observed TB risk in guselkumab‐treated patients suggests IL‐23 inhibitors may be better treatment options than TNF inhibitors in high‐risk patients, including those in TB‐endemic regions. Consistent with other studies [2, 3, 5, 6, 7, 8], these findings, along with increased liver transaminases in patients who received preventive treatment (especially isoniazid) and potential for hepatotoxicity, support the recently published NPF‐IPC consensus to remove requirements for TB screening in most circumstances for patients initiating an IL‐23 inhibitor for psoriatic disease.

## Funding

This work was supported by Johnson and Johnson.

## Ethics Statement

All studies were conducted in accordance with the ethical principles that have their origin in the Declaration of Helsinki and that are consistent with Good Clinical Practice. For all studies, the protocol was approved by an institutional review board or independent ethics committee at each site. Participants in all studies provided written informed consent. All 11 studies are registered with ClinicalTrials.gov with the following identifiers: NCT01483599, NCT02207231, NCT02207244, NCT02203032, NCT02905331, NCT03090100, NCT02325219, NCT02319759, NCT03162796, NCT03158285, and NCT03796858.

## Conflicts of Interest

L.P. has received consultancy/speaker's honoraria from and/or participated in clinical trials sponsored by AbbVie, Almirall, Amgen, Biogen, Boehringer Ingelheim, Bristol Myers Squibb, Fresenius‐Kabi, Horizon (DSMB), Johnson & Johnson, LEO Pharma, Eli Lilly, Novartis, Pfizer, Samsung‐Bioepis, STADA, Sun Pharma, and UCB. T.‐F.T. has received consulting fees for AbbVie, AnaptysBio, Boehringer Ingelheim, Bristol Myers Squibb, Celgene, Eli Lilly, Galderma, GlaxoSmithKline, Johnson & Johnson, LEO Pharma, Merck Sharp & Dohme, Novartis, Pfizer, PharmaEssentia, Sanofi, Sun Pharma, and UCB. E.R.S. has served as a consultant for AbbVie, Johnson & Johnson, Novartis, and Roche; received grant/research support from AbbVie, Johnson & Johnson, Novartis, Pfizer, Roche, and UCB; and speaker fees from Amgen, Bristol Myers Squibb, Eli Lilly, Johnson & Johnson, Novartis, Pfizer, Roche, and UCB. T.B. is currently a principal investigator for studies being sponsored by AbbVie, Castle, CorEvitas, Dermavant, Galderma, Mindera, and Pfizer. She has additional research funding from Novartis and Regeneron. She has served as an advisor for AbbVie, Arcutis, Boehringer Ingelheim, Bristol Myers Squibb, Eli Lilly, Johnson & Johnson, LEO Pharma, Pfizer, Novartis, Sun Pharma, and UCB. M.M., A.P.K., S.L., H.L., S.O., J.P., and E.A. are employees of Johnson & Johnson; employees may own stock and/or stock options. M.G. has been a speaker, investigator, or advisory board member for AbbVie, Acelyrin, Alumis, Amgen, Akros, AnaptysBio, Apogee, Arcutis, Arena, Aslan, Bausch Health, Boehringer Ingelheim, Bristol Myers Squibb, Coherus, Dermira, Dermavant, Eli Lilly, Galderma, GlaxoSmithKline, Incyte, InMagene, JAMP Pharma, Johnson & Johnson, Kyowa Kirin, LEO Pharma, Medimmune, Merck, Moonlake, Nimbus, Novartis, Pfizer, Regeneron, Reistone, Sanofi Genzyme, Sun Pharma, Tarsus, Takeda, Union, UCB, and Vyne. A.L.G. has received consulting fees from AbbVie, Boehringer Ingelheim, Bristol Myers Squibb, Eli Lilly, Galderma, Johnson & Johnson, LEO Pharma, Novartis, Pfizer, and Sanofi. M.G.L. is an employee of Mount Sinai and receives research funds from AbbVie, Arcutis, Avotres, Boehringer Ingelheim, Bristol Myers Squibb, Cara Therapeutics, Clexio, Dermavant, Eli Lilly, Incyte, Inozyme, Johnson & Johnson, Oruka, Pfizer, Sanofi‐Regeneron, and UCB, and is a consultant for AbbVie, Added Health, Aikium, Almirall, AltruBio, Alumis, Amgen, Apogee, Arcutis, AstraZeneca, Atomwise, Avotres, Boehringer Ingelheim, Bristol Myers Squibb, Castle, Celltrion, CorEvitas, Dermavant, Dermsquared, Edsea, Eli Lilly, Evommune, Facilitation of International Dermatology Education, Forte Biosciences, Galderma, Genentech, Incyte, Johnson & Johnson, LEO Pharma, Mayne, Meiji Seika, Mindera Health, Mirum, MoonLake, Novartis, Oruka, Pfizer, Revolo, Sanofi‐Regeneron, Seanergy, Strata, Sun Pharma, Takeda, Trevi, and Verrica. P.N. has received funding for research and clinical trials and honoraria for advice and lectures on behalf of AbbVie, Amgen, Boehringer Ingelheim, Bristol Myers Squibb, Celgene, Eli Lilly, Galapagos, GlaxoSmithKline, Johnson & Johnson, Novartis, Pfizer, Sun Pharma, and UCB.

## Data Availability

The data sharing policy of Johnson & Johnson is available at https://innovativemedicine.jnj.com/our‐innovation/clinical‐trials/transparency. As noted on this site, requests for access to the study data can be submitted through Yale Open Data Access Project site at http://yoda.yale.edu.
